# Non-Medical Switching or Discontinuation Patterns among Patients with Non-Valvular Atrial Fibrillation Treated with Direct Oral Anticoagulants in the United States: A Claims-Based Analysis

**DOI:** 10.3390/jmahp12030020

**Published:** 2024-09-02

**Authors:** Michael Ingham, Hela Romdhani, Aarti Patel, Veronica Ashton, Gabrielle Caron-Lapointe, Anabelle Tardif-Samson, Patrick Lefebvre, Marie-Hélène Lafeuille

**Affiliations:** 1 Janssen Scientific Affairs LLC—A Johnson & Johnson Company, Titusville, PA 08560, USA; 2 Analysis Group Inc., Montréal, QC H3B 0G7, Canada

**Keywords:** anticoagulant, atrial fibrillation, discontinuation, insurance, non-medical switching, socioeconomic factors, treatment switching

## Abstract

This study assessed direct-acting oral anticoagulant (DOAC) switching/discontinuation patterns in patients with non-valvular atrial fibrillation (NVAF) in 2019, by quarter (Q1–Q4), and associated socioeconomic risk factors. Adults with NVAF initiating stable DOAC treatment (July 2018–December 2018) were selected from Symphony Health Solutions’ Patient Transactional Datasets (April 2017–January 2021). Switching/discontinuation rates were reported in 2019 Q1–Q4, separately. Non-medical switching/discontinuation (NMSD) was defined as the difference between switching/discontinuation rates in Q1 and mean rates across Q2–Q4. The associations of socioeconomic factors with switching/discontinuation were assessed. Of 46,793 patients (78.7% ≥ 65 years; 52.6% male; 7.7% Black), 18.0% switched/discontinued their initial DOAC in Q1 vs. 8.8% on average in Q2–Q4, corresponding to an NMSD of 9.2%. During the quarter following the switch/discontinuation, more patients who switched/discontinued in Q1 remained untreated (Q1: 77.0%; Q2: 74.3%; Q3: 71.2%) and fewer reinitiated initial DOAC (Q1: 17.6%; Q2: 20.8%; Q3: 24.0%). Factors associated with the risk of switching/discontinuation in Q1 were race, age, gender, insurance type, and household income (all *p* < 0.05). More patients with NVAF switched/discontinued DOACs in Q1 vs. Q2–Q4, and more of them tended to remain untreated relative to those who switched/discontinued later in the year, suggesting a potential long-term impact of NMSD. Findings on factors associated with switching/discontinuation highlight potential socioeconomic discrepancies in treatment continuity.

## 1. Background

Atrial fibrillation (AF) is a highly prevalent form of cardiac arrhythmia that is associated with increased mortality resulting from complications such as stroke and thromboembolism [1]. AF currently affects at least 3 to 6 million people in the United States (US) [1], with related strokes accounting for over 15% of all strokes [2]. Non-valvular AF (NVAF) is the most common type of AF [3].

Long-term anticoagulation therapy is critical for the prevention of stroke and thromboembolic complications in patients with NVAF [4,5]. In the past decade, direct-acting oral anticoagulants (DOACs) have become increasingly recommended as the first-line treatment for NVAF [4,5,6]. Relative to conventional vitamin K antagonists such as warfarin, DOACs have been associated with superior or non-inferior efficacy and better safety profiles in clinical trials and in routine practice [7,8]. Accordingly, DOACs are recommended over warfarin in AF guidelines published by multiple organisations, including the American Heart Association/American College of Cardiology/Heart Rhythm Society (AHA/ACC/HRS) [4], the American College of Chest Physicians (CHEST) [5], the Canadian Cardiovascular Society (CCS) [9], the European Society of Cardiology (ESC) [10], and the European Stroke Organisation (ESO) [11]. While DOACs have been increasingly adopted for NVAF management [12], changes to DOAC treatment such as switching or discontinuation remain common [13,14].

A change to a prescribed anticoagulation therapy may be due to medical reasons (e.g., lack of clinical efficacy or response, side effects) or non-medical reasons (e.g., cost, insurance coverage) [13,15]. For example, a change in a stable patient’s prescription medication for non-medical reasons may be due to formulary changes implemented by insurers aiming at decreasing the acquisition costs of prescription medications [16]. Formulary changes may include the designation of preferred formulary medications, the exclusion of certain medications, or the addition of new restrictions for a given medication [17]. In fact, formulary designs per se (e.g., re-starting of deductibles or re-defining of cost-sharing levels) may create incentives for non-medical treatment changes [18]. As a result, some patients may lose access to their medication, face increased out-of-pocket costs for it, or cease treatment altogether, leading to non-medical switching or discontinuation (NMSD) [17,19].

Considerable evidence suggests that NMSD, particularly with medications for chronic diseases, may pose a negative impact on patient outcomes [16,17,19,20,21,22,23]. In a survey conducted by the American Society for Preventive Cardiology among 254 patients with cardiovascular disease whose anticoagulant was non-medically switched by payers, 22% of patients reported resurfacing symptoms, 28% reported side effects after the switch, and 20% discontinued treatment as a result [19]. Systematic reviews have also found that NMSD is associated with subsequent increase in healthcare resource use and altered medication-taking behaviour, including poorer adherence and persistence [16,20].

While prior studies have evaluated switching or discontinuation rates of patients with NVAF treated with DOACs in the US [24,25,26], to the best of our knowledge, no real-world study has focused on NMSD in this population. This study aimed to describe DOAC switching and discontinuation patterns in 2019, by quarter, in patients with NVAF recently stabilised on their initial DOAC treatment prior to the start of 2019, and to estimate the proportion of switching or discontinuation due to potential non-medical reasons. Specifically, given that most formulary changes typically happen in the beginning of a calendar year [27], along with insurance plan deductibles reset that could result in greater out-of-pocket expense required earlier in the year [28], the incremental switching or discontinuation rate of initial DOAC treatment in the first quarter (Q1) vs. subsequent quarters of 2019 among patients who were stable on their initial DOAC treatment at the start of 2019 was used as a proxy to estimate the rate of NMSD in this study. Furthermore, as disparities in DOAC treatment and the impact of cost-sharing strategies have been reported [29,30], potential demographic and socioeconomic factors associated with switching or discontinuation of initial DOAC treatment in Q1 were also evaluated.

## 2. Materials and Methods

### 2.1. Data Source

Data from Symphony Health Solutions’ Patient Transactional Datasets, covering the period of 1 April 2017, to 1 January 2021, were used. This open claims data source contains patient demographics, medical and procedure claims, and prescription drug claims. Open claims capture a patient’s healthcare activity regardless of whether the patient maintains the same healthcare plan, as long as the patient uses providers from the network that supplies data to the database. The database captures > 75% of all US retail prescription claims, representing over three-quarters of the US population annually across multiple payer channels (i.e., commercial, Medicare, Medicaid); the remaining < 25% of claims are outside of the covered networks. Data were de-identified and comply with the patient requirements of the Health Insurance Portability and Accountability Act; therefore, no review by an institutional review board was required per Title 45 of CFR, Part 46.101(b)(4).

### 2.2. Study Design

This study used a retrospective observational design. Continuous clinical activity started at the time of the first pharmacy or medical claim and ended when a gap of ≥3 months between two subsequent claims was observed. Stable treatment with a DOAC was defined as having ≥2 approved claims for the DOAC agent and no gap in treatment > 60 days [31].

### 2.3. Patient Selection

Patients were included in the study sample if (1) they had ≥1 final approved claim (i.e., submitted by a pharmacy and approved for payment by health plans after claims adjudication) for a DOAC used for NVAF (i.e., apixaban, dabigatran, rivaroxaban, or edoxaban), with initial DOAC treatment starting on or after 1 July 2018; (2) they had continuous clinical activity period starting ≥ 6 months before the first DOAC claim (i.e., washout period) and lasting at least until the end of 2019; (3) they had ≥1 claim with a diagnosis of AF (International Classification of Diseases, 10th Revision, Clinical Modification code: I48) during the washout period; (4) they were ≥18 years old on the date of the first DOAC claim; and (5) they were stable on their initial DOAC treatment at the start of 2019. Patients were excluded if (1) they had ≥1 claim for a condition associated with the use of anticoagulation treatment other than NVAF (including mitral stenosis, mechanical heart valve, venous thromboembolism, and hip or knee replacement surgery), or ≥1 claim for an organ or tissue transplant, during the washout period; or (2) they were pregnant at any time during or after the washout period.

### 2.4. Outcomes and Variables

Baseline demographic and socioeconomic characteristics, including age, gender, ethnicity, insurance type, household income, education, and region of residence, were reported.

Modifications in treatment were assessed in 2019 and included treatment switch defined as initiation of an oral anticoagulant (OAC) among apixaban, dabigatran, rivaroxaban, edoxaban, or warfarin that was different from the initial DOAC; and discontinuation of initial DOAC defined as a gap in treatment of >60 days [31], with the date of discontinuation defined as the day following the end of the stable episode on the initial DOAC after 1 January 2019. In addition, treatment patterns following the switching or discontinuation event, including proportions of patients remaining untreated, reinitiating the initial DOAC, and initiating a new OAC different from the initial DOAC, were assessed in 2019.

Non-medical switching or discontinuation (i.e., NMSD) was defined as the difference between the rate of switching or discontinuation of initial DOAC observed in Q1 of 2019 and the mean rate of switching or discontinuation observed across the second (Q2), third (Q3), and fourth (Q4) quarters of 2019. This was based on the assumption that most formulary and health plan design changes happen at the beginning of a calendar year [27], and hence treatment switching or discontinuation in Q2 to Q4 could be less likely driven by these non-medical reasons compared to Q1.

### 2.5. Statistical Analysis and Visualisation

Baseline demographic and socioeconomic characteristics, as well as patients with switching or discontinuation events, were described using frequencies and proportions. The occurrence of switching or discontinuation of initial DOAC events by quarter of 2019 was visualised using a Sankey diagram. The rate of NMSD was reported descriptively. A second Sankey diagram was created to visualise the treatment patterns in quarters following the switching or discontinuation event among patients who switched or discontinued the initial DOAC in Q1, Q2, and Q3 of 2019, separately.

The demographic and socioeconomic factors associated with switching or discontinuation of initial DOAC in Q1 were assessed using multivariable logistic models, adjusting for time (in months) since initiation of initial DOAC. Factors associated with switching or discontinuation of initial DOAC were reported using odds ratios (ORs), along with the 95% confidence intervals and *p*-values.

## 3. Results

### 3.1. Study Sample Characteristics

A total of 46,793 patients were stable on their initial DOAC treatment at the beginning of 2019 and were included in the study. Among them, 78.7% were ≥65 years and 52.6% were male. Most of the patients were white (66.2%), and less than one-tenth were black (7.7%). Almost a third of the sample (31.7%) had commercial insurance, and the others had non-commercial insurance (Medicare: 61.4%; Medicaid: 6.4%; other: 0.5%). Household income was <$50,000 in 30.7% of the patients, ≥$100,000 in 19.8%, and unknown in 18.1%. The US region of residence was South in 40.1% of the patients (Table 1). Baseline characteristics of patients who switched or discontinued their initial DOAC anytime in 2019 and those who switched or discontinued their initial DOAC anytime in Q1 are presented in Supplementary Table S1.

### 3.2. Switching and Discontinuation Patterns

**Figure 1:**
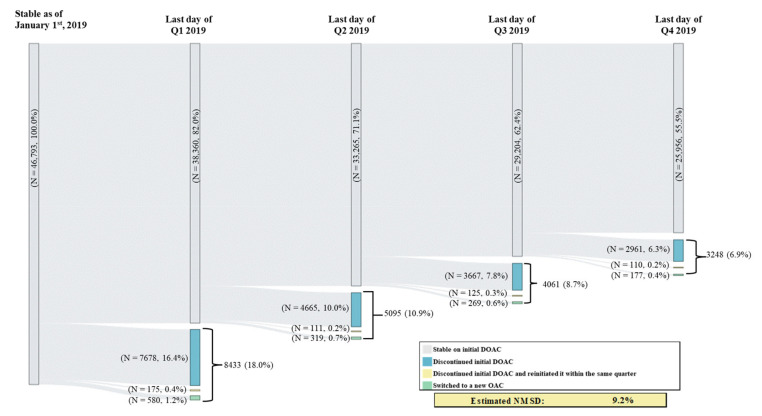
Switching or Discontinuation of Initial DOAC in 2019 by Quarter. DOAC = direct-acting oral anticoagulant, OAC = oral anticoagulant, NMSD = non-medical switching or discontinuation; Q1 = first quarter, Q2 = second quarter, Q3 = third quarter, Q4 = fourth quarter.

**Figure 2:**
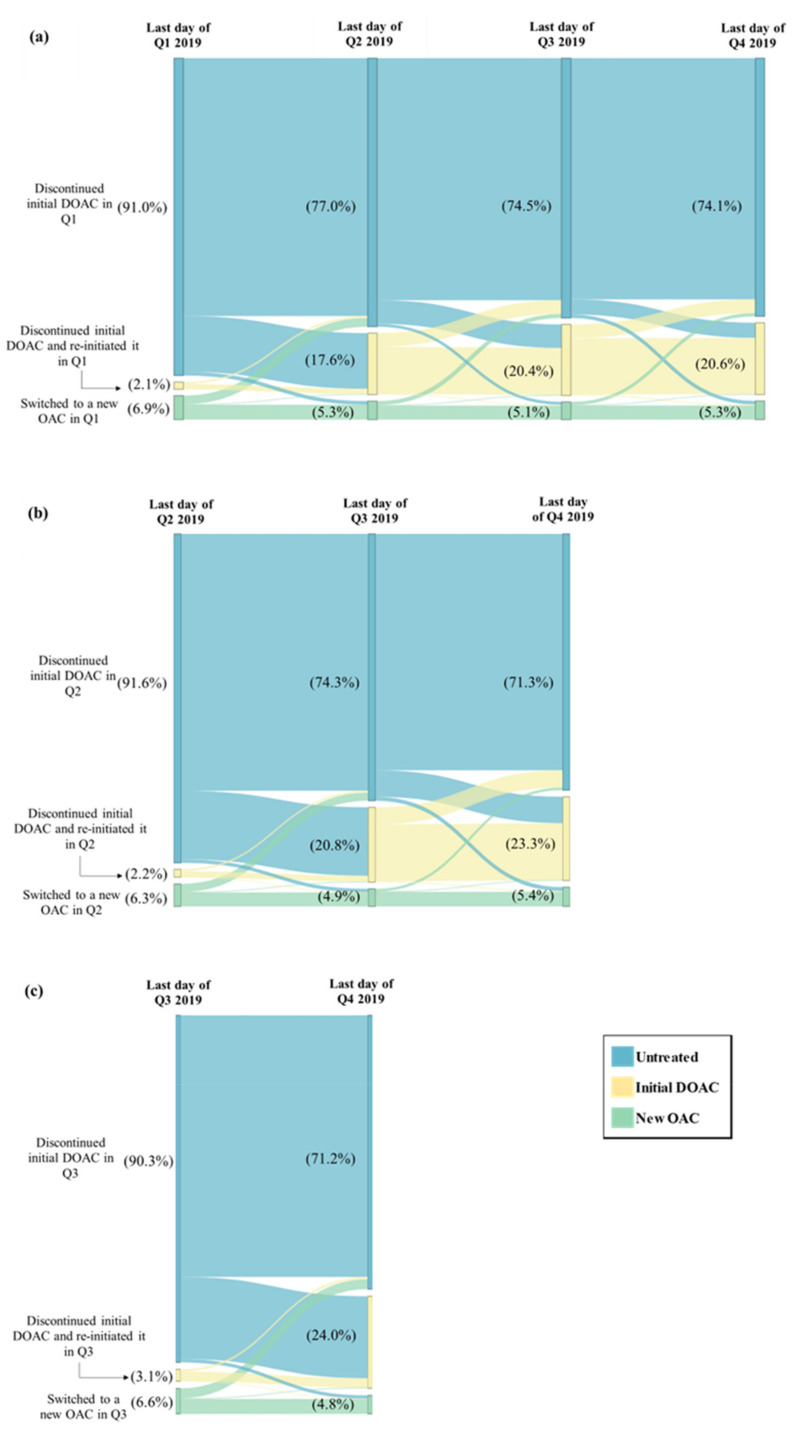
Treatment Patterns Following the Switching or Discontinuation Event Among Patients Who Switched or Discontinued in (**a**) Q1 (N = 8433), (**b**) Q2 (N = 5095), and (**c**) Q3 (N = 4061). DOAC = direct-acting oral anticoagulant, OAC = oral anticoagulant, Q1 = first quarter, Q2 = second quarter, Q3 = third quarter, Q4 = fourth quarter.

Among patients in the study sample, 18.0% had switched or discontinued their initial DOAC in Q1 vs. an average of 8.8% across the other quarters of 2019 (switching or discontinuation rate in Q2: 10.9%; Q3: 8.7%; Q4: 6.9%); this difference in switching or discontinuation rate in Q1 vs. the average rate across Q2 to Q4 corresponds to an estimated percentage of NMSD equal to 9.2% (Figure 1). Among patients with a switching or discontinuation of the initial DOAC event occurring in Q1, 77.0% remained untreated during the entire subsequent quarter (i.e., Q2); this percentage was numerically lower among patients with the event occurring in Q2 (74.3% remained untreated during Q3) and those with the event occurring in Q3 (71.2% remained untreated during Q4) (Figure 2). In addition, fewer patients with a switching or discontinuation of the initial DOAC event occurring in Q1 reinitiated the initial DOAC during the subsequent quarter (17.6% reinitiated the initial DOAC in Q2), as compared to those with the event in Q2 (20.8% reinitiated the initial DOAC in Q3) or in Q3 (24.0% reinitiated the initial DOAC in Q4) (Figure 2).

### 3.3. Demographic and Socioeconomic Factors Associated with Switching or Discontinuation of Initial DOAC

**Figure 3:**
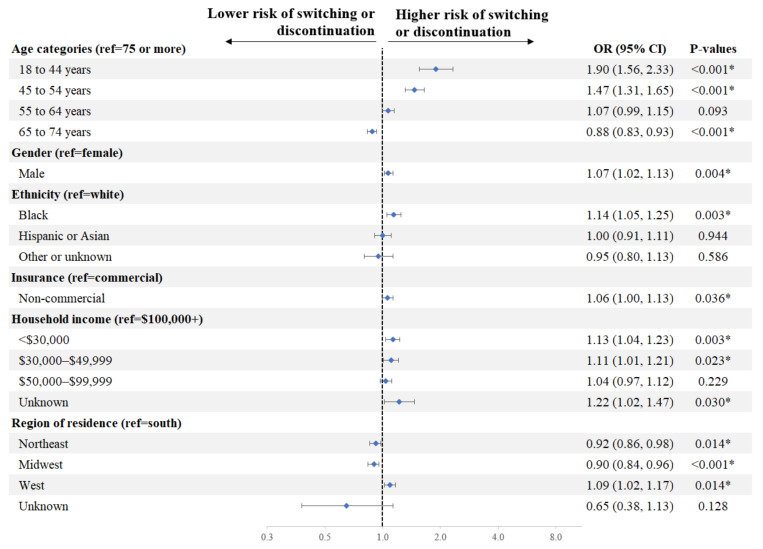
Factors Associated with Switching or Discontinuation of Initial DOAC in Q1. * Indicates a *p*-value < 0.05. Education was not included in the model given its strong correlation with household income. CI = confidence interval, DOAC = direct-acting oral anticoagulant, OR = odds ratio, ref = reference.

Factors associated with a significantly higher risk of switching or discontinuation of initial DOAC in Q1 included age (18–44 vs. ≥75 years: OR = 1.90, *p* < 0.001; 45–54 vs. ≥75 years: 1.47, *p* < 0.001), race (Black vs. White: OR = 1.14, *p* = 0.003), household income (<$30,000 vs. $100,000+: OR = 1.13, *p* = 0.003; $30,000–$49,999 vs. $100,000+: OR = 1.11, *p* = 0.023), gender (male vs. female: OR = 1.07, *p* = 0.004), and insurance type (non-commercial vs. commercial: OR = 1.06, *p* = 0.036) (Figure 3).

## 4. Discussion

In this large retrospective claims-based analysis among patients with NVAF recently stabilised on their initial DOAC treatment in 2019, a considerably higher proportion of patients were observed to have switched or discontinued their initial DOAC in Q1 (18.0%) relative to subsequent quarters (8.8% on average). Although there is a lack of literature evaluating reasons for switching or discontinuing DOAC at various timepoints of the year, it stands to reason that the rate of DOAC switching or discontinuation due to medical reasons such as treatment efficacy and safety should be constant throughout the year, particularly among patients who are already stable on their medication. Hence, the current observation suggests that non-medical factors could have played a role in the higher switching or discontinuation rate in Q1. One of such factors could be that deductible reset in Q1 together with copay amounts may place financial barriers to treatment continuation [28,32], and hence resulting in more NMSD, especially for patients with commercial insurance [18]; as the year progresses, patients are more likely to reach deductible and/or maximum copay limits, which reduce their financial disincentives to NMSD [28]. On this premise, NMSD was estimated in this study at 9.2% (i.e., the difference in switching or discontinuation rate in Q1 vs. the average rate across Q2 to Q4), representing approximately half (i.e., 9.2%/18.0%) of all switching or discontinuation of initial DOAC observed in Q1. Given the high prevalence of NVAF and increasing use of DOAC in the US [1,3,12], a substantial volume of patients with NVAF could be impacted by NMSD despite the seemingly low switching or discontinuation rates observed. Furthermore, more patients who had a switching or discontinuation event in Q1 tended to remain untreated, and fewer appeared to reinitiate the initial DOAC or to switch to a new OAC in the remainder of the year as compared to those who had an event later in the year, suggesting the potential negative long-term impact of NMSD on treatment continuity.

The mean switching or discontinuation rate in Q2 to Q4 in the current study appears consistent with the average discontinuation rate of DOAC among patients newly diagnosed with AF in US clinical practice, which has been reported at 9.5% at 6 months in a multicenter registry study [25]; thus, the considerably higher switching or discontinuation rate of DOAC in Q1 may likely be attributable to factors unique to this quarter. Q1 coincides with the timing of formulary changes that are generally implemented by insurers at the beginning of a calendar year [27]. As a cost-containment strategy, a formulary change may force patients to switch to a lower-cost alternative or face increased out-of-pocket costs to continue with their original medication that is no longer on the formulary; patients who prefer not to try new medications but cannot afford the increased costs may opt to abandon their well-tolerated treatment [17,23]. Furthermore, the deductible and copay expenses earlier in the year may also contribute to the lower tendency for patients to fill prescriptions [28,32], as cost-related concerns have been reported as a major factor impacting patients’ treatment decisions and medication-taking behaviours [33,34]. Importantly, evidence has suggested that NMSD such as those arising from formulary changes and high-deductible health plans can pose a negative impact on patients’ clinical and economic outcomes [16,20,35,36]. Therefore, policymakers should comprehensively evaluate the consequences of NMSD when designing benefit plans, as these may impact patient outcomes.

The current study also found that younger age, Black race, and lower income were associated with a significantly higher risk of switching or discontinuation in Q1, which largely aligns with previous findings of disparities in DOAC treatment and persistence patterns in these populations [29,37,38]. For instance, younger age and lower socioeconomic status have been shown to be risk factors for poor DOAC treatment persistence in the literature [37,39,40]; therefore, patients with these risk factors may be at a higher risk of switching or discontinuing DOAC in general, including for non-medical reasons. Notably, patients of Black race and of lower socioeconomic status have been shown to be disproportionately affected by high cost-sharing strategies imposed by insurers [30,41,42]. For instance, a US study among enrollees insured in high-deductible plans found that plan members of low socioeconomic status had reduced inpatient and high-severity emergency department visits during their first enrolment year, potentially an attempt to minimise out-of-pocket expenses, but the need for acuity care increased in the following year; these trends were not observed among plan members with high socioeconomic status [30]. Together, these findings underscore the need to consider socioeconomic factors in assessing the consequences of cost-sharing strategies and non-medical switching in the NVAF population to help prevent further widening of health disparities.

The findings of this study should be interpreted with limitations. First, the database does not capture services received from providers outside of the network; thus, the use of pharmacological treatments and medical services could be underestimated. Second, as with all claims or transactions database studies, prescription fills do not account for whether the medication dispensed was taken as prescribed. Third, as reason for discontinuation was not available in the data, NMSD was defined based on proxy measures, which were not validated; the estimated NMSD rate may not have captured all NMSD events and may also have encompassed switching and discontinuation events due to medical reasons. Nonetheless, our study design aimed to minimise the potential for including these events due to medical reasons, as we followed the same group of stable patients throughout the study and assessed the within-patient change by quarter, thereby minimizing confounding factors such as patients’ clinical conditions and improving the reliability of our findings. Future investigations, including replication of the analyses in other calendar years and comparisons of switching and discontinuation rates specifically due to formulary changes, are warranted. Fourth, the analysis of the association between socioeconomic factors and switching/discontinuation in Q1 did not control for clinical characteristics such as risk scores, but these characteristics may have an impact on switching or discontinuation of DOAC. For instance, prior studies have suggested that patients with NVAF are less likely to discontinue oral anticoagulants if they are older, with congestive heart failure, hypertension, diabetes, and prior stroke/transient ischaemic attack [39,43]. Since these factors are correlated with age [44,45], and we controlled for age in the socioeconomic factor analysis, these factors could have been at least partially controlled for. Finally, this study may have been subjected to residual confounding due to unmeasured confounders (i.e., information not available or underreported in transaction data, such as the cause of AF, patient-provider discussions, requirements for monitored therapy, and presence of side effects).

## 5. Conclusions

This large retrospective claims-based analysis found that patients with NVAF recently stabilised on their initial DOAC treatment tended to switch or discontinue more frequently in Q1 vs. other quarters, potentially driven by non-medical reasons such as formulary changes early in the year and benefit plan design elements. More patients who switched or discontinued their initial stable DOAC treatment in Q1 tended to remain untreated compared to those who did so later in the year, suggesting a potential long-term impact of NMSD. Younger, Black, and lower-income patients were more likely to switch from or discontinue their initial DOAC in Q1, highlighting potential socioeconomic discrepancies in treatment continuity. Policymakers should comprehensively evaluate the potential consequences of NMSD, including consideration of socioeconomic factors, when designing benefit plans and cost-sharing elements for the NVAF population.

## Figures and Tables

**Table 1:** Baseline Characteristics of Patients with NVAF Initiated on DOAC.

	N = 46,793
**Age categories, N (%)**	
18 to 44 years	474 (1.0)
45 to 54 years	1841 (3.9)
55 to 64 years	7665 (16.4)
65 to 74 years	14,549 (31.1)
75 years or more	22,264 (47.6)
**Gender, N (%)**	
Male	24,617 (52.6)
Female	22,176 (47.4)
Ethnicity, N (%)	
White	30,982 (66.2)
Black	3623 (7.7)
Hispanic or Asian	2716 (5.8)
Other or unknown	9472 (20.2)
**Insurance, N (%)**	
Non-commercial	31,946 (68.3)
Medicare	28,736 (61.4)
Medicaid	2980 (6.4)
Other	230 (0.5)
Commercial	14,847 (31.7)
**Household income, N (%)**	
<$30,000	8273 (17.7)
$30,000–$49,999	6091 (13.0)
$50,000–$99,999	14,674 (31.4)
$100,000+	9272 (19.8)
Unknown	8483 (18.1)
**Education, N (%)**	
High school or less	10,680 (22.8)
Some college	16,651 (35.6)
Associate degree and above	10,940 (23.4)
Unknown	8522 (18.2)
**Census region of residence, N (%)**	
Northeast	9942 (21.2)
Midwest	11,037 (23.6)
West	6946 (14.8)
South	18,760 (40.1)
Unknown	108 (0.2)
**Month of initiation in 2018, N (%)**	
Jul	10,105 (21.6)
Aug	11,190 (23.9)
Sep	10,869 (23.2)
Oct	13,259 (28.3)
Nov	1370 (2.9)

DOAC = direct oral anticoagulant, NVAF = non-valvular atrial fibrillation.

## Data Availability

The data that support the findings of this study are available from Symphony Health Solutions. Restrictions apply to the availability of these data, which were used under license for this study.

## Supplementary Materials

The following supporting information can be downloaded at: https://www.mdpi.com/article/10.3390/jmahp12030020/s1, Table S1: Demographic Characteristics of Patients with NVAF Initiated on DOAC who Discontinued in 2019.

## Abbreviations

AF	Atrial fibrillation
DOAC	Direct-acting oral anticoagulant
NMSD	Non-medical switching or discontinuation
NVAF	Non-valvular atrial fibrillation
OAC	Oral anticoagulant
OR	Odds ratio
US	United States
