# Supplementary material for: Non-Medical Switching or Discontinuation Patterns among Patients with Non-Valvular Atrial Fibrillation Treated with Direct Oral Anticoagulants in the United States: A Claims-Based Analysis
Source: J Mark Access Health Policy. 2024 Sep 2;12(3):252–63. doi: 10.3390/jmahp12030020 (PMC11417902; doi:10.3390/jmahp12030020)
Supplement: Supplementary file 1 [file jmahp-12-00020-s001.zip › jmahp-3024029-supplementary.pdf]

Article

Non-Medical Switching or Discontinuation Patterns Among Patients with Non-Valvular Atrial Fibrillation Treated with Direct Oral Anticoagulants in the United States: A Claims-Based Analysis – Supplementary Table

Supplementary Table S1. Demographic Characteristics of Patients with NVAF Initiated on DOAC who Discontinued in 2019

	Discontinued or switched in 2019 N = 20,837	Discontinued or switched in Q1 2019 N = 8433
**Age categories, N (%)**		
18 to 44 years	294 (1.4)	142 (1.7)
45 to 54 years	970 (4.7)	450 (5.3)
55 to 64 years	3413 (16.4)	1435 (17.0)
65 to 74 years	6081 (29.2)	2363 (28.0)
75 years or more	10,079 (48.4)	4043 (47.9)
**Gender, N (%)**		
Male	11,227 (53.9)	4536 (53.8)
Female	9610 (46.1)	3897 (46.2)
**Ethnicity, N (%)**		
White	13,375 (64.2)	5402 (64.1)
Black	1782 (8.6)	736 (8.7)
Hispanic or Asian	1284 (6.2)	497 (5.9)
Other or unknown	4396 (21.1)	1798 (21.3)
**Insurance, N (%)**		
Non-commercial	14,532 (69.7)	5770 (68.4)
Medicare	13,018 (62.5)	5150 (61.1)
Medicaid	1409 (6.8)	577 (6.8)
Other	105 (0.5)	43 (0.5)
Commercial	6305 (30.3)	2663 (31.6)
**Household income, N (%)**		
<$30,000	3906 (18.7)	1569 (18.6)
$30,000–$49,999	2852 (13.7)	1127 (13.4)
$50,000–$99,999	6297 (30.2)	2538 (30.1)
$100,000+	3805 (18.3)	1566 (18.6)
Unknown	3977 (19.1)	1633 (19.4)
**Education, N (%)**		
High school or less	4908 (23.6)	1946 (23.1)

Some college	7308 (35.1)	2910 (34.5)
Associate degree and above	4630 (22.2)	1934 (22.9)
Unknown	3991 (19.2)	1643 (19.5)
**Census region of residence, N (%)**		
Northeast	4114 (19.7)	1708 (20.3)
Midwest	4640 (22.3)	1862 (22.1)
West	3317 (15.9)	1376 (16.3)
South	8719 (41.8)	3472 (41.2)
Unknown	47 (0.2)	15 (0.2)
**Month of initiation in 2018, N (%)**		
Jul	4210 (20.2)	1678 (19.9)
Aug	4929 (23.7)	1946 (23.1)
Sep	4804 (23.1)	1935 (22.9)
Oct	6241 (30.0)	2615 (31.0)
Nov	653 (3.1)	259 (3.1)

DOAC = direct oral anticoagulant; NVAF = non-valvular atrial fibrillation
